# Sign realized jump risk and the cross-section of stock returns: Evidence from China's stock market

**DOI:** 10.1371/journal.pone.0181990

**Published:** 2017-08-03

**Authors:** Youcong Chao, Xiaoqun Liu, Shijun Guo

**Affiliations:** 1 Business School, Central South University, Changsha, Hunan, P. R. China; 2 Tourism School, Hainan University, Haikou, Hainan, P. R. China; 3 Business School, The University of Queensland, St. Lucia, Brisbane, Australia; University of Rijeka, CROATIA

## Abstract

Using 5-minute high frequency data from the Chinese stock market, we employ a non-parametric method to estimate Fama-French portfolio realized jumps and investigate whether the estimated positive, negative and sign realized jumps could forecast or explain the cross-sectional stock returns. The Fama-MacBeth regression results show that not only have the realized jump components and the continuous volatility been compensated with risk premium, but also that the negative jump risk, the positive jump risk and the sign jump risk, to some extent, could explain the return of the stock portfolios. Therefore, we should pay high attention to the downside tail risk and the upside tail risk.

## Introduction

With the development of financial econometrics, the measurement of financial risk variables has made a major breakthrough, which makes the study on time series and cross-sectional stock return-risk relations revealed by the Capital Asset Pricing Model (CAPM) model and the three-factor model has achieved great success. Whether risk measures, such as conditional skewness, tail risk, jumps and rare disaster risk, have a certain risk premium and the similarities and differences among them have become a main focus within the academic circle.

In the frame of the GARCH-class model based on low-frequency data, the short-run and long-run components of market risk have different contributions for the cross-sectional pricing of the volatility risk, and the short-run components capture market skewness risk, which is interpreted as a measure of the tightness of financial constraints[1].Chang estimates a conditional and forward-looking implied market skewness of market returns from the daily Standard & Poor’s 500 index option data, and the results show that the market skewness risk premium is statistically and economically significant and cannot be explained by other common risk factors, such as the market excess return or the size, book-to-market, momentum, and market volatility factors, or by firm characteristics[2]. Chan and Feng find that time-varying jump risk premia are significantly associated with extreme news events or jumps in four stock market index futures returns[3]. Furthermore, there is a new perspective to investigate the return- (high order) risk trade-off relationship under the macro-economic framework with time-varying uncertainties. Some researchers deem that the extended long-run risk model (Extended LRR) that embeds time-varying jumps in the model in various ways (learning, model uncertainty, etc.) can measure the risk premium[4,5]. Kelly and Jiang propose a bivariate prediction equation with the tail risk factors and macro-economic variables [6], which can be derived by the LRR model[7] and the Disaster Risk model[8]. It can be seen that jumps affect the equity premium in indirect ways; they obtained the estimated jump value through the options, or the jump was embedded in the conditional variance, conditional skewness, kurtosis and other conditions of higher moments of dynamic equations.

Under the framework of high-frequency data with a nonparametric method, the presence of jumps in stocks, portfolios and indexes has been confirmed[9–11]. A number of studies directly estimate the jump intensity, jump mean, and jump volatility and use those realized jump measures as proxies for the entire realized jump risk, rather than as the proxy of skewness, to illustrate its explanatory power on the cross-sectional asset returns[12–15]. Furthermore, Bollerslev and Todorov estimated the jump tail based on the new Extreme Value Theory and high frequency data and combined the jump tail with the investors’ expectation of their fear of extreme events that may occur, proving that the time-varying compensation needed for the expectation of a disaster can explain large parts of the average equity premium and variance risk premium[9]. Another attraction for using a nonparametric method with high frequency data to identify the realized jump variables is that this method can calculate the positive and negative jumps and their distributions in a simple yet accurate way. In regard to stock price movements, large negative price changes, which occur to a certain, large enough extent, would cause much greater future volatility compared to a positive price change of the same magnitude[16]. Further, Guo et al. considered the difference between the upside and downside risks, named the sign jump volatility, to investigate the possible influence on the index return; the empirical results showed that sign jump risk is an important determinant of equity premium, and this risk factor needs to be priced[17].

To gain insights into the existing studies, we find that all the empirical analysis only use either the index or individual stocks for a time series regression to probe into whether skewness, tail risk, rare disaster risk and realized jump could predict future returns. However, skewness, rare disaster and jump are tails in the theory of statistics, so it is important to distinguish positive and negative tails. Therefore, from the perspective of the portfolio level, the paper, on the cross-sectional level, further investigates whether positive, negative realized jumps and sign realized jumps could predict the excess portfolio return with the Fama-MacBeth two-step regression test. According to the theoretical relations between extreme positive, negative returns and the asymmetric character of volatility, the non-parametric method with high frequency data could be used to make accurate estimation to positive and negative realized jump variables representing upside and downside risk, which will shed new light on our understanding and grasp of financial market rules.

The remainder of the paper is organized as follows. Section 2 describes the identification method for the high frequency positive, negative and sign realized jumps and introduces the jump risk factor model that we employ, and Section 3 discusses the high-frequency data that we study and presents descriptive statistics. Empirical results are presented in Section 4, and we conclude in Section 5.

## Methods and materials

### Estimation of realized jumps

While jumps are known to be very crucial in asset pricing, the estimation of jump components by the parametric model has been questioned for stability over different sample time periods. Recently, realized variance utilizing the information in the intraday data have been used to measure and forecast volatilities[18,19]. More recently, Barndorff-Nielsen and Shephard proposed a series in their seminal work on bi-power variation measure used to divide the realized variance into continuous diffusion volatility and jump volatility, which make different contributions to capturing the dynamic characteristics of the volatility model[20,21].

Under the reasonable presumption that jumps in financial markets are usually rare and large, we assume that there is at most one jump per day and that the jump size dominates the daily return when a jump occurs. These assumptions allow us to extract the daily realized jumps and to explicitly calculate the monthly jump intensity, size, and standard deviation.

Let *p*_*t*_ = *log*(*P*_*t*_) denote the time *t* logarithmic price of the asset, and assume that it evolves in continuous time as a jump-diffusion process:

$$dp_{t}=\mu_{t}\mathrm{dt}+\sigma_{t}dW_{t}+J_{t}dq_{t}$$ (1)

Where *dq*_*t*_ is a Poisson jump process with intensity *λ*_*J*_, and *J*_*t*_ is the corresponding log jump size distribution following a normal distribution *N*(*μ*_*J*_, *σ*_*J*_). Consider

$${\mathrm{RV}}_{t}=\sum_{j=1}^{M}r_{t,j}^{2}\to \int_{t\text{-}1}^{t}\sigma_{s}^{2}\mathrm{ds}+\int_{t\text{-}1}^{t}J_{s}^{2}dq_{s}$$ (2)

where *M* is the total number of trades between time *t* and *t* + 1, *j* is the indication for each trade, *RV*_*t*_ is the realized volatility at time t and *r*_*t*,*j*_ *= lnP*_*t*,*j*_*-lnP*_*t*,*j-1*_. In the realistic financial markets, the price volatility of financial asset is not continuous but contains jumps due to the influence aroused by information shocks on the market. To measure discrete jump variation (*RJV*), a method called realized bipower variation (*BV*) is proposed[21,22]:

$${\mathrm{BV}}_{t}\equiv \frac{\pi }{2}\frac{M}{M\text{-}1}\sum_{j=2}^{M}\left|r_{t,j}||r_{t,j\text{-}1}\right|\to \int_{t\text{-}1}^{t}\sigma_{s}^{2}\mathrm{ds}$$ (3)

Where $\sqrt{\pi /2}=Ε(\Pi_{t})$, Π_t_ is a standardized normal distribution random variable, and *M*/(*M-1*) is the amendment to sample size. According to Barndorff-Nielsen and Shephard, the difference between *RV*_*t*_ and *BV*_*t*_ is just the consistent estimator of the discrete jump variation when *M*→∞; that is,

$${\mathrm{RV}}_{t}\text{-}{\mathrm{BV}}_{t}\overset{M\to \infty }{\to }{\mathrm{RJV}}_{t}$$ (4)

To enhance the accuracy of discrete jump variation, the *Z*_*t*_ statistic based on the bipower variation theory is used to identify the discrete jump variation[21,23,24]. *Z*_*t*_ is given as follows:

$$Z_{t}=\frac{\frac{{\mathrm{RV}}_{t}\text{-}{\mathrm{BV}}_{t}}{{\mathrm{RV}}_{t}}}{\sqrt{\left(\frac{\pi^{2}}{4}+\pi \text{-}5)\frac{1}{M}\max(1,\frac{{\mathrm{TP}}_{t}}{{\mathrm{BV}}_{t}^{2}}\right)}}$$ (5)

Where,

$${\mathrm{TP}}_{t}=\frac{M}{M\text{-}2}∙\frac{M}{4{\left[\Gamma (7/6)/\Gamma (1/2)\right]}^{3}}∙\sum_{i=3}^{M}{\left|r_{t,i}\right|}^{4/3}{\left|r_{t,i\text{-}1}\right|}^{4/3}{\left|r_{t,i\text{-}2}\right|}^{4/3}$$ (6)

Under the significance level, 1−*α*, we obtain the estimation of the discrete jump variation:

$${\mathrm{RJV}}_{t}=I(Z_{t}>\Phi_{\alpha }^{\text{-}1})\sqrt{\left[{\mathrm{RV}}_{t}\text{-}{\mathrm{BV}}_{t}\right]}$$ (7)

where *Φ* is the cumulative distribution function of a standard normal, and $I_{t,\alpha }=I(Z_{t}\geq \Phi_{\alpha }^{\text{-}1})$ is the resulting indicator function for whether there is a jump during the day; $I_{t,\alpha }=I(Z_{t}\geq \Phi_{\alpha }^{\text{-}1})$ equals 1 when a jump is detected on day *t* and equals 0 otherwise. In the process of actual operation, we need to choose an appropriate *α*; previous studies revealed that when jump contributions are 10% and 80%, the significance level should be 0.99 and 0.999, respectively[12].

With the above test of the *Z*_*t*_ statistic and the related bipower variation theory, we obtain the estimators of *BV*_*t*_ and *RJV*_*t*_ and then calculate the monthly jump size (*Size_RJV*), monthly jump size mean (*Mean_RJV*), monthly jump arrival rate (*Arr_RJV*), and monthly jump size standard deviation (*Std_RJV*). The jump components are defined as follows:

$${\mathrm{Size\_RJV}}_{\mathrm{month}}=\sum {\mathrm{Size\_RJV}}_{\mathrm{day}}$$ (8)

$${\mathrm{Mean\_RJV}}_{\mathrm{month}}={\mathrm{Size\_RJV}}_{\mathrm{month}}/\mathrm{N\_RJV\_days}$$ (9)

$${\mathrm{Arr\_RJV}}_{\mathrm{month}}=\mathrm{N\_RJV\_days}/\mathrm{days}$$ (10)

$${\mathrm{Std\_RJV}}_{\mathrm{month}}={\left[\sum \left({\left({\mathrm{Size\_RJV}}_{\mathrm{day}}\text{-}{\mathrm{Mean\_RJV}}_{\mathrm{day}}\right)}^{2}\right)\right]}^{1/2}$$ (11)

Where *N_RJV days* is the total number of days when a realized jump occurs, and “days” denotes the trading days in a month. We follow previous studies and set the confidence level *α* at 0.95 in this paper[12,15].

### Construction of empirical model

The realized negative (positive) semi-variance can distinguish downside volatility risk from upside volatility risk. Based on this method, the signed jump variation (difference between the upside risk and the downside risk) is further defined as containing continuous volatility and positive or negative jump variation[22,25,26], but our sign jump variation is the difference between the positive jump volatility and the negative jump volatility.

When estimating the realized jump volatility and extracting its characteristic components, we assume that there is at most one jump in a trading day, which has a dominant effect on the stock return once it occurs, and this means that extreme changes in the stock returns are accompanied by the presence of jumps. The non-parametric method can not only identify jump volatility and estimate distribution parameters but can also calculate monthly jump factors accurately and easily, including the sum of monthly positive and negative jumps, monthly jump intensity, monthly jump mean, monthly jump arrival rate and monthly jump standard deviation, which help depict extreme changes in the returns and verify the asymmetric characteristic of volatility more effectively. At this time,

$$P_{t}\mathrm{\_Size\_RJV}=I\{r_{t}\geq 0\}\mathrm{Size\_}{\mathrm{RJV}}_{t}$$ (12)

$$N_{t}\mathrm{\_Size\_RJV}=I\{r_{t}\leq 0\}\mathrm{Size\_}{\mathrm{RJV}}_{t}$$ (13)

$${\mathrm{Sign}}_{t}\mathrm{\_Size\_RJV}=P_{t}\mathrm{\_Size\_RJV-}N_{t}\mathrm{\_Size\_RJ}$$ (14)

Correspondingly, we are able to get the positive, negative and sign jump mean, arrival rate, and the standard deviation, expressed as, *P*_*t*__*Mean*,*P*_*t*__*Arr*, *P*_*t*__*Std*, *N*_*t*__*Mean*, *N*_*t*__*Arr*,*N*_*t*__*Std*, *Sign*_*t*__*Mean*,*Sign*_*t*__*Arr*, *Sign*_*t*__*Std*, respectively.

Meanwhile, based on the simple linear relations between jump components [12,13], we use similar methods as Bail and Peng, who used the lagged one month realized jump variation components as the predictive value of the conditional variance[27], that is, the regression model is constructed as shown below:

$$r_{i,t+1}\text{-}r_{f,t}=\gamma_{0t}+\gamma_{1t}{\mathrm{Mean\_RJV}}_{i,t}+\gamma_{2t}{\mathrm{Arr\_RJV}}_{i,t}+\gamma_{3t}{\mathrm{Std\_RJV}}_{i,t}+\gamma_{4t}{\mathrm{BV}}_{i,t}+\varepsilon_{i,t+1}$$ (15)

Based on this model, we consider the negative and positive jump volatility components and sign jump volatility. Thus, the following three equations are constructed as follows, while equation Eq (16) and Eq (17) consider only the negative and positive jump components, respectively, and equation Eq (18) includes only the factor of sign jump components, Eq (19) and Eq (20) expand Eq (16), Eq (17) and Eq (18) to portray the significance of the sign jump factors with the control of the negative or positive jump factors.

$${r_{i,t}\text{-}r_{f,t}=\gamma }_{\mathrm{ot}}+\gamma_{1t}{\mathrm{N\_Mean}}_{i,t\text{-}1}+\gamma_{2t}{\mathrm{N\_Arr}}_{i,t\text{-}1}+\gamma_{3t}{\mathrm{N\_Std}}_{i,t\text{-}1}+\gamma_{4t}{\mathrm{BV}}_{i,t\text{-}1}+\varepsilon_{i,t}$$ (16)

$${r_{i,t}\text{-}r_{f,t}=\gamma }_{\mathrm{ot}}+\gamma_{1t}{\mathrm{P\_Mean}}_{i,t\text{-}1}+\gamma_{2t}{\mathrm{P\_Arr}}_{i,t\text{-}1}+\gamma_{3t}{\mathrm{P\_Std}}_{i,t\text{-}1}+\gamma_{4t}{\mathrm{BV}}_{i,t\text{-}1}+\varepsilon_{i,t}$$ (17)

$${r_{i,t}\text{-}r_{f,t}=\gamma }_{\mathrm{ot}}+\gamma_{1t}{\mathrm{Sign\_Mean}}_{i,t\text{-}1}+\gamma_{2t}{\mathrm{Sign\_Arr}}_{i,t\text{-}1}+\gamma_{3t}{\mathrm{Sign\_Std}}_{i,t\text{-}1}+\gamma_{4t}{\mathrm{BV}}_{i,t\text{-}1}+\varepsilon_{i,t}$$ (18)

$${r_{I,t}\text{-}r_{f,t}=\gamma }_{\mathrm{ot}}+\gamma_{1t}{\mathrm{N\_Mean}}_{I,t\text{-}1}+\gamma_{2t}{\mathrm{N\_Arr}}_{I,t\text{-}1}+\gamma_{3t}{\mathrm{N\_Std}}_{I,t\text{-}1}+\gamma_{4t}{\mathrm{Sign\_Mean}}_{I,t\text{-}1}+\gamma_{5t}{\mathrm{Sign\_Arr}}_{i,t\text{-}1}+\gamma_{6t}{\mathrm{Sign\_Std}}_{i,t\text{-}1}+\gamma_{7t}{\mathrm{BV}}_{i,t\text{-}1}+\varepsilon_{i,t}$$ (19)

$${r_{i,t}\text{-}r_{f,t}=\gamma }_{\mathrm{ot}}+\gamma_{1t}{\mathrm{P\_Mean}}_{i,t\text{-}1}+\gamma_{2t}{\mathrm{P\_Arr}}_{i,t\text{-}1}+\gamma_{3t}{\mathrm{P\_Std}}_{i,t\text{-}1}+\gamma_{4t}{\mathrm{Sign\_Mean}}_{i,t\text{-}1}+\gamma_{5t}{\mathrm{Sign\_Arr}}_{i,t\text{-}1}+\gamma_{6t}{\mathrm{Sign\_Std}}_{i,t\text{-}1}+\gamma_{7t}{\mathrm{BV}}_{i,t\text{-}1}+\varepsilon_{i,t}$$ (20)

It is noteworthy that explanatory variables of Eq (15) represent the following four different combinations: *Mean_RJV*, *Arr_RJV*, *Std_RJV* or *Size_RJV*, *Arr_RJV*, *Std_RJV* or *Size_RJV*, *Mean_RJV*, *Std_RJV* or *Size_RJV*, *Mean_RJV*, *Std_RJV*; during the actual operation of 84 successive months with the OLS regressions, these four combinations are considered separately, and by comparing the adjusted goodness of fit, squared residuals and other indicators, and conducting the overall simulation of 84 months, we determine which of these combinations are more reasonable.

### Data set

Different sampling frequencies of intraday data can affect the research results. On the one hand, low sampling frequency may fail to depict the actual volatility information on that day. On the other hand, high sampling frequency may lead to the problem of micro-noise, which may affect the results. As suggested by previous studies[21,24], five-minute high-frequency data are used for return calculation. The data was collected from the CSMAR high-frequency database from January 4, 2007 to December 31, 2013 for all A-share stocks traded in China’s stock market. The trading hours of China’s stock market span two sessions on a trading day: a morning session from 9:30am to 11:30am and an afternoon session from 1:00pm to 3:00pm. At most, 49 transactions can be collected per day (including one overnight transactions and 48 intraday transactions), and there are 84 months’ data in total. The value-weighted monthly returns of 25 portfolios were obtained directly from the RESSET database. We calculate all monthly jump components with high-frequency transactions and run the regression on monthly return over monthly jump components.

### Construction of the investment portfolios

In the past, researchers mainly used the composite index or individual stocks with trading activity, larger market capitalization to study the dynamic characteristics of the realized jump volatility, and its model-predictive[9,10]; one possible reason is that the calculation of the jump process for the dynamic portfolios is complex and cumbersome based on the index and individual stocks. However, for the return-risk trade-off, on the one hand, although the index has been largely used as the sample, it is hard to arrive at a robust conclusion for the individual stocks, which is sensitive to the presence of micro-noise; on the other hand, more importantly, in addition to the time series dimensions, cross-sectional data are indispensable for complete research. In fact, 25 portfolios constructed in the Fama-French model laid a theoretical and empirical economic foundation for the future portfolio-based empirical analysis. It is of great economic application significance to explore whether the realized jump risk may predict the excess portfolios return, which is a salutary supplement of the index from the market level and individual stocks from a firm level.

In this paper, based on the Fama-French three-factor asset pricing model, we use the 25 portfolios that are determined by the size and book-to-market ratio and their constituent stocks as a testing asset. The group forming strategy is as follows: the size is market value at the end of June of year T, the book-to-market is the book value of equity in fiscal year T-1 divided by market value at the end of December, T-1 year; we take the respective quintiles of the market value and the book-to-market, then we obtain 25 groups at the intersection of the two quintiles and finally, according to these portfolios, we calculate their monthly market capitalization-weighted average returns from July, T year to June, T+1 year.

Portfolios with constituent stocks are time-varying every year from 2007 to 2013, that is, in fact, there are 7 * 25 = 175 sets of data. The daily realized jump intensity, mean, standard deviation and arrival rate for each stock are calculated based on five-minute high frequency data of each constituent stock in each group of 25 portfolios per year. Then, all 12 of the monthly jump data for each stock are simply additive combinations of the corresponding daily jump data; finally, the monthly jump components for this portfolio are the average of all constituent stocks. Synthesizing all the data of 7 years, every jump component of each portfolio has 12 monthly jump data every year; then, we obtain 84 sets of time series data of the four jump components for each portfolio. It is worth noting that time varying constituent stocks of the 25 portfolios are not what matter; it is the fact that s1b1…s1b5, s2b1…s2b5,…, s5b1…s5b5 as a whole achieve their significance. Therefore, the constituent stocks that calculate the jump values of all 25 portfolios in 2007 are different from those in 2008 as well as in the seventh year, 2013, as well. By analogy, although different stocks constitute the specified portfolios every year, they can still reflect the time-varying realized jump volatility of the 25 portfolios in 7 years.

## Results and discussions

### Summary statistics of portfolios and jumps

Panel A of Table 1 reports the mean, variance, and Sharpe ratio of the market portfolio in the sample interval; group B shows the mean, variance, and Sharpe ratio of the 25 portfolios, which were selected based on their market value and book value; s1-s5 represent the market values from low to high, and b1-b5 are the book-to market ratios from low to high, i.e., s5b5 represents the equity portfolio with the largest market value and the highest book-to-market ratio. As shown, the mean returns of all 25 portfolios are higher than the market portfolio, and 22 portfolios’ Sharpe ratio is larger than that of the market portfolio.

**Table 1:** Descriptive statistic for portfolio return.

Portfolios	Mean	Standard Deviation	Sharp Ratio	Portfolios	Mean	Standard Deviation	Sharp Ratio
**Panel A. Returns of the Market Asset Portfolio**							
Mkt	0.0013	0.0903	0.0869				
**Panel B. Returns of the 25 Portfolios Constructed by the Size and the B/M**							
s1b1	0.0320	0.1251	0.2391	s4b1	0.0108	0.1148	0.0756
s1b2	0.0308	0.1246	0.2299	s4b2	0.0225	0.1250	0.1633
s1b3	0.0256	0.1215	0.1936	s4b3	0.0174	0.1219	0.1251
s1b4	0.0393	0.1328	0.2799	s4b4	0.0199	0.1207	0.1473
s1b5	0.0351	0.1240	0.2660	s4b5	0.0163	0.1173	0.1208
s2b1	0.0249	0.1333	0.1711	s5b1	0.0115	0.1183	0.0795
s2b2	0.0283	0.1273	0.2059	s5b2	0.0162	0.1130	0.1247
s2b3	0.0276	0.1237	0.2061	s5b3	0.0110	0.1281	0.0697
s2b4	0.0234	0.1157	0.1842	s5b4	0.0137	0.1126	0.1028
s2b5	0.0267	0.1236	0.1993	s5b5	0.0122	0.0972	0.1040
s3b1	0.0228	0.1235	0.1672				
s3b2	0.0210	0.1211	0.1559				
s3b3	0.0280	0.1297	0.1997				
s3b4	0.0174	0.1167	0.1306				
s3b5	0.0191	0.1208	0.1408				

**Fig 1:**
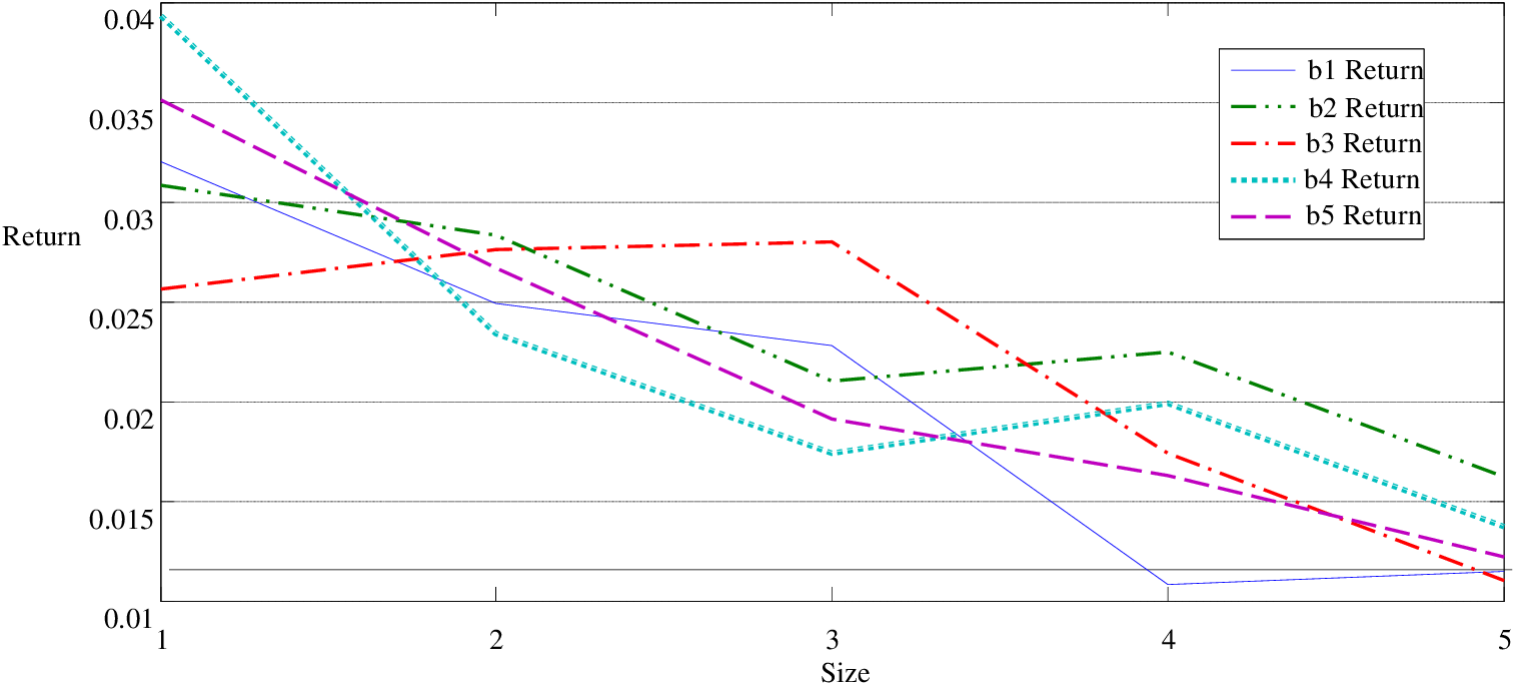
Return of Size effect with Fixed B/M *_*bi. When b1, b2, b3, b4 and b5 are respectively controlled, the returns of portfolios are decreasing with the increasing size.

**Fig 2:**
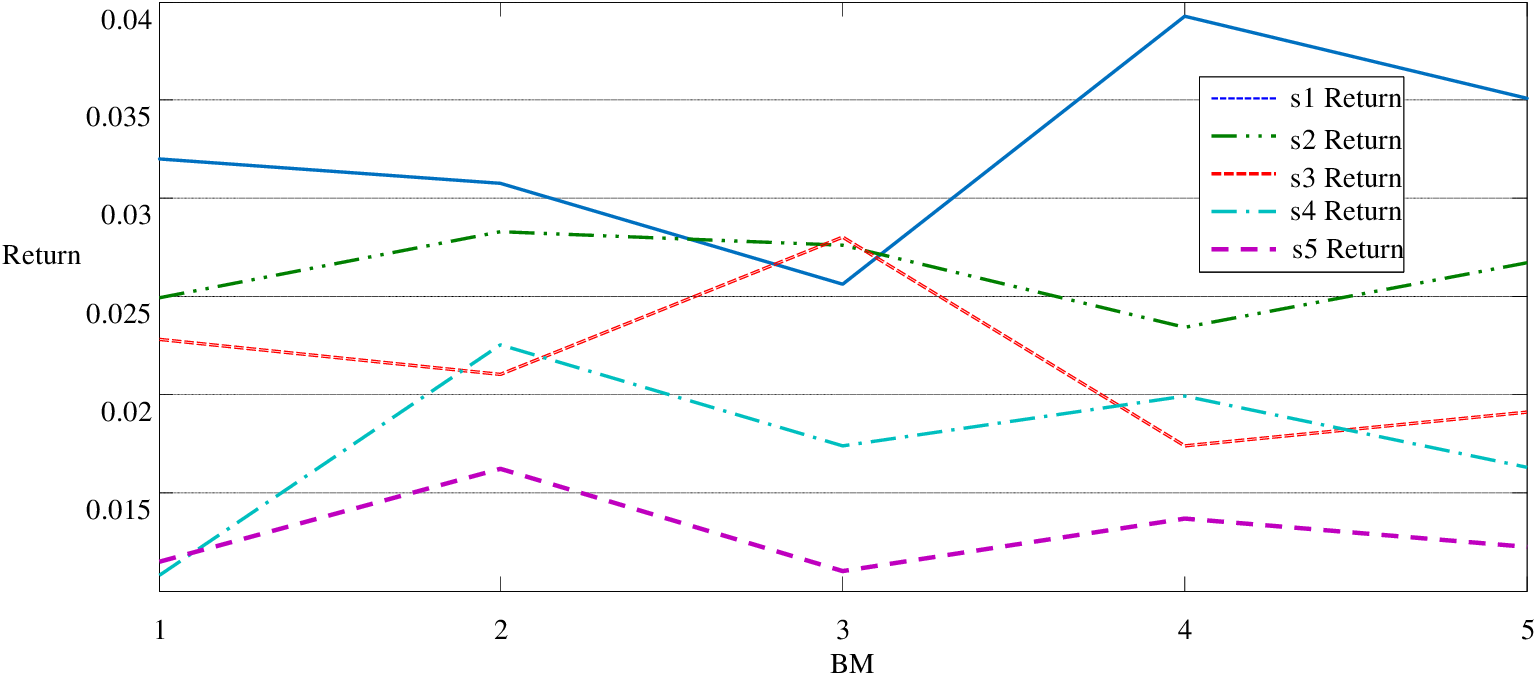
Return of BM effect with Fixed Size_si. When s1, s2, s4 and b5 are respectively controlled, the return of the highest BM portfolio is higher than the lowest BM portfolio. Note that the positive relationship between BM and returns does not appear when s3 is under control.

Panel B of Table 1 and Figs 1 and 2 show the average returns of China's A-share market portfolios for seven years is consistent with the results of Fama and French[28]. It indicates that the average return of stock portfolio has a reverse correlation with the firm size and has a positive correlation with the book-to-market ratio.

As shown in Fig 1 and panel B of Table 1, under control of the smallest B/M value b1, the return of the smallest size portfolio s1b1 is 0.0320 higher than other 4 portfolios; in detail, the return of s2b1, s3b1, s4b1 and s5b1are 0.0249, 0.0228, 0.0108 and 0.0115, respectively. Likewise, when b2, b3, b4 and b5 are respectively controlled, the returns of portfolios are decreasing with the increasing size. Similarly, see from Fig 2 and Panel B of Table 1, when size is under control, the return of the highest BM portfolio s1b5 is higher than the lowest BM portfolio s1b1, when s1 is under control. The same results are found when s2, s4 or s5 is controlled. Note that the positive relationship between BM and returns does not appear when s3 is under control, and the effect of BM on portfolios’ return is not strictly increased with the increasing BM value, which is different from the size effect.

Table 2 reports the portfolios’ average positive and negative realized jump components in the sample interval, including the jump size (*P (N)_Size*), mean (*P (N)_Mean*), arrival rate (*P (N)_Arr*) and standard deviation mean (*P (N)_Std*) (Only results of s1b1-s1b5 are presented here; see other results in Tables A-D in S1 Text). It is obvious that the positive and negative realized jump components series have significant characteristics of “leptokurtosis and heavy tails”. Meanwhile, the number of positive jumps (*NP*) is larger than the number of negative ones (*NN*) for each portfolio. In this case, although the impact of negative shocks is higher than the positive shocks, the occurrence of the former has a lower probability. Consequently, the positive jump intensity and other positive jump components are higher than those of the negative jumps. We also find that the means of the sign realized jump intensities for all portfolios are positive and appear as the property “high peak and fat tail”(see S1 Table).

**Table 2:** Descriptive statistics of the jump components for s1b1-s1b5.

**Panel A Descriptive Statistics of the Positive Jump Components**							
**Portfolios**	**NN**	**NP**	**N_Size**	**N_Mean**	**N_Arr**	**N_Std**	**BV**
s1b1	1.1001***	2.9642***	0.0233***	0.0136***	0.0557***	0.0113***	0.0221***
s1b2	1.1924***	2.9022***	0.0259***	0.0146***	0.0603***	0.0120***	0.0238***
s1b3	1.2059***	2.8771***	0.0292***	0.0162***	0.0613***	0.0132***	0.0260***
s1b4	1.2401***	2.9857***	0.0307***	0.0171***	0.0630***	0.0141***	0.0271***
s1b5	1.2413***	3.2091***	0.0300***	0.0169***	0.0629***	0.0139***	0.0282***
**Panel B Descriptive Statistics of the Negative Jump Components**							
**Portfolios**	**NN**	**NP**	**P_Size**	**P_Mean**	**P_Arr**	**P_Std**	**BV**
s1b1	1.1001***	2.9642***	0.0595***	0.0195***	0.1488***	0.0028***	0.0221***
s1b2	1.1924***	2.9022***	0.0628***	0.0203***	0.1463***	0.0032***	0.0238***
s1b3	1.2059***	2.8771***	0.0689***	0.0223***	0.1463***	0.0038***	0.0260***
s1b4	1.2401***	2.9857***	0.0729***	0.0226***	0.1526***	0.0037***	0.0271***
s1b5	1.2413***	3.2091***	0.0837***	0.0239***	0.1647***	0.0039***	0.0282***

***, **, * represent the 1%, 5%, and 10% significance levels of the Jarque-Bera Test, respectively.

### Fama-MacBeth cross-sectional regressions

For each portfolio, we make its excess return the dependent variable and its jump volatility factors and the continuous volatility the explanatory variables to do a time series regression analysis with the 84 sets of time series data, and we obtain the corresponding coefficients of these risks premium factors. Then, we calculate the means and standard deviations of the above sets of coefficients, namely, *r*_*kt*_(*k = 0*,*1*,*2*,*3*,*4*), which are the explanatory variables in the second step of the Fama-MacBeth cross-sectional regression, and all 84 monthly average excess returns are the dependent variables. T-statistics are used to test whether the jump volatility components can significantly affect the cross-sectional returns of the portfolios.

First, for all 84 months, we conduct regression analysis with Eq (15) and Eq (16) to Eq (20) six times each. Only the 12-month results indicate that the jump volatility components do not significantly affect the cross-sectional returns of the portfolios for only 12 months, while the results of the remaining 72 months are significant, which confirms that jump components can explain and predict the cross-sectional returns of the 25 portfolios with different combinations of negative, positive, and sign jump volatility. Table 3 provides the summary of the number of significant months for the 25 portfolios.

**Table 3:** Significant number of months with different regression equations.

Regression Equations	Significant Months
Significant Months for Jump (Eq 15)	40 Months in Total
Significant Months for Negative Jump (Eq 16)	34 Months in Total
Significant Months for Positive Jump (Eq 17)	43 Months in Total
Significant Months for Sign Jump (Eq 18)	41 Months in Total
Significant Months for Sign and Negative Jump (Eq 19)	55 Months in Total
Significant Months for Sign and Positive Jump (Eq 20)	42 Months in Total

Next, as shown in Table 4, for Eq (15), out of the 84 months, there are 29 months whose jump components explain the excess return in the form of a linear combination of Mean, Arr and Std, and there are 22 months whose explanatory variables are the linear combination of Size, Mean and Std; additionally, there are 21 months in the form of a linear combination of Size, Arr and Std. More generally, the majority of the jump components’ coefficients are positive, which is in accord with the economic theory of high risk with high risk premium. There are also 8 months whose continuous volatility (BV) is significant; in general, the negative coefficient of BV is always accompanied by the negative coefficients of the Arr and Std, and there are also positive coefficients of the Mean and Std. For those negative jump and continuous volatility values, it is possible to interpret them from the perspective of the negative relationship between idiosyncratic volatility and return.

**Table 4:** Regression results under different linear combination of the jump components.

Linear Combinations	Size	Arr	Std	BV	Number of Significant Months
Size / Arr/ Std	4+, 4-	6+, 5-	7+, 5-	3+, 2-	21
P_ Size / Arr/ Std	7+, 2-	4+, 6-	3+, 8-	1+, 2-	19
N_ Size / Arr/ Std	3+, 4-	3+, 4-	3+, 3-	1+, 2-	14
Sign_ Size /Arr / Std	8+, 4-	8+, 7-	3+, 8-	5+, 4-	24
P_Sign_ Size / Arr / Std	—	—	—	—	22
N_Sign_ Size/ Arr / Std	—	—	—	—	31
	**Size**	**Mean**	**Std**	**BV**	
Size / Mean/ Std	5+, 3-	3+, 6-	7+, 4-	1+, 2-	22
P_ Size / Mean/ Std	11+, 3-	7+, 4-	2+, 5-	2+, 2-	23
N_ Size / Mean/ Std	3+, 8-	8+, 2-	2+, 7-	1+, 1-	13
Sign_ Size / Mean/ Std	6+, 2-	3+, 6-	6+, 4-	2+, 1-	20
P_Sign_Size/ Mean/ Std	—	—	—	—	13
N_Sign_Size/ Mean/ Std	—	—	—	—	24
	**Size**	**Arr**	**Mean**	**BV**	
Size / Arr / Mean	4+, 2-	4+, 6-	5+, 5-	1+, 3-	18
P_ Size / Arr / Mean	7+, 0-	2+, 6-	2+, 6-	1+, 2-	15
N_ Size / Arr / Mean	2+, 4-	2+, 4-	4+, 3-	1+, 1-	13
Sign_ Size / Arr / Mean	7+, 7-	7+, 8-	4+, 5-	4+, 4-	21
P_Sign_ Size / Arr/ Mean	—	—	—	—	19
N_Sign_ Size / Arr/ Mean	—	—	—	—	20
	**Mean**	**Arr**	**Std**	**BV**	
Mean / Arr / Std	8+, 5-	9+, 7-	7+, 6-	1+, 3-	29
P_Mean/ Arr / Std	7+, 6-	9+, 9-	2+, 6-	3+, 1-	26
N_Mean/ Arr / Std	4+, 8-	3+, 8-	8+, 3-	2+, 2-	18
Sign_Mean/ Arr / Std	1+, 3-	6+, 4-	4+, 5-	2+, 4-	25
P_Sign_Mean/ Arr / Std	—	—	—	—	22
N_Sign_Mean/ Arr / Std	—	—	—	—	31

The symbols “+” and “-” represent the positive and negative coefficients of the variables, respectively.

Third, as for Eq (16) and Eq (17), the linear combinations of *P_Mean*, *P_Arr* and *P_Std* and *N_Mean*, *N_Arr* and *N_Std* have the largest number of significant months, 26 and 18, respectively. There are both negative and positive coefficients of the negative or the positive jump components, and the numbers of negative coefficients of the negative jump mean and arrival rate for the negative jump components are larger than for the corresponding positive coefficients; the ratios are 8/4 and 8/3, respectively. However, the numbers of positive coefficients of the positive jump mean, standard deviation and arrival rate are larger than for the corresponding negative coefficients, which suggest that part of the increased current volatility comes from the negative or positive jumps of the past month. Under the four linear combinations of the negative and positive jump components, the numbers of months with significant continuous volatility (BV) are 14 and 11, respectively, and the coefficients are half positive and half negative. The jump volatility and the continuous volatility are both significant in the regression model, which indicates that the two types of volatility make different contributions to explaining the excess return of the portfolios. Meanwhile, for some portfolios, their jump component factors are significant and yet their continuous volatility is non-significant, which means that it is necessary to add the extreme jump volatility risk to the equilibrium analysis due to different cross-sectional characteristics when we discuss the relationship between return and risk. As is seen from the results, different types of linear combinations of the jump components should be considered when we study the effect of jump volatility on the excess return of the portfolio. Through the analysis and comparison of the different jump components’ combinations, the optimal linear portfolio regression with the highest value of goodness-of fit and the least MSE could be found.

Furthermore, according to the studies concerning sign realized jump and positive and negative realized jump means[13,17], in this paper, the sign jump components in terms of a certain linear combination are directly treated as the explanatory variables using the same regression method as Eq (15), Eq (16) and Eq (17) to explore whether these factors contain some additional information for predicting the stock return. Eq (18) considers only the sign jump variables, Eq (19) and Eq (20) test the robustness of the sign jump factor under the control of the positive and negative jump components. In terms of the economic theory, not only the asymmetric characteristic of the volatility or the theory of leverage but also the loss-aversion of prospect theory can explain the asymmetry: the reduction in the subjective utility of a unit loss is greater than the increase in the utility of the same amount of gain. The explanation is verified by empirical analysis, which finds that the mean of the positive jump components is larger than the mean of the negative ones [26].

The empirical results show that there are 43 and 34 months for which positive and negative jumps are significant, respectively, and 41 months for which the sign jumps are significant, which is almost the same as the situation when the linear jump combination is only considered, for which the number of significant months is 42. In addition, the number of significant months is 42 when the positive jump and the sign volatility are both considered, while the number of significant months reaches 55 when the negative jump and the sign volatility are added into the regression. The results demonstrate that both the negative and positive jumps are the common factors that can explain and predict the excess returns; in particular, the negative jumps have greater explanatory power when the sign jump factors are under control. There is no significant law for the positive or negative value of the coefficients of the significant jump variables, but it might be a problem deserving of systematic search that the sign of the same variable is different under different linear combinations.

Finally, all the above 84 coefficients of the jump components and continuous volatility are used as explanatory variables for the second step of the Fama-MacBeth cross-sectional regression, and the average excess returns of all months through seven years are treated as explained variables. The significance of the coefficients of these variables is tested by T-statistics or P-Values.

The results are shown in Table 5: For Eq (19), whose explanatory variables are the linear combination of *N_Sign_Size*, *N_Sign_Arr*, *N_Sign _Std* and *BV*, the sign jump arrival rate passes the t-statistic test, which refutes the null hypothesis of a zero coefficient at a significance level of 10%, and the coefficient is significantly negative. In addition, as for Eq (18), the linear combination of *Sign_Size*, *Sign_Arr*, *Sign_Std* and *BV* is used to conduct OLS regression, and the coefficients of the sign jump intensity and the realized continuous volatility are significantly positive at the 5% significance level, which indicates that both the sign jump risk and the continuous volatility risk obtain risk compensation. According to Eq (15), the regression results demonstrate that the realized standard deviation has a negative risk price, which can be interpreted by the highly positive correlation between the idiosyncratic volatility risk and the jump risk, and the idiosyncratic volatility has a negative risk price. In Eq (18), the coefficient for *Sign_Size* has a statistically significant mean of 0.0330 (*t* = -2.0146). The results strongly support that sign jump components are priced and indicate that the cross-sectional returns are monotonically increasing with sign jump size.

**Table 5:** Cross-sectional regression results.

Panel A. Results of Eq (19)		Panel B. Results of Eq (18)		Panel C. Results of Eq (15)	
Variables	Coefficients	Variables	Coefficients	Variables	Coefficients
Intercept	0.0155 (1.1345)	Intercept	0.0125 (0.9667)	Intercept	0.0195 (1.5399)
N_Size	-0.0041 (-0.7276)	Sign_Size	0.0330** (2.0146)	Mean	0.0033 (0.9471)
N_Arr	-0.0222* (-1.7815)	Sign_Arr	-0.0043 (-0.1543)	Arr	-0.0528 (-1.5944)
N_Std	-0.0032 (-0.8817)	Sign_Std	0.0062 (1.4514)	Std	-0.0022** (-2.0074)
Sign_Size	0.0109 (0.7663)	BV	0.0084** (1.9053)	BV	0.0054 (1.3892)
Sign_Arr	-0.0145 (-0.6161)				
Sign_Std	0.0023 (0.6011)				
BV	0.0046 (1.1958)				
Adj. R^2^	0.0284	Adj. R^2^	0.0339	Adj. R^2^	0.0613

This table reports the results of cross-sectional regressions of monthly portfolio returns in each month. t-statistics are shown in parentheses.

***, **, * represent the 1%, 5%, and 10% significance levels, respectively.

The above results shed light on the complex way in which jump risks perform; other possible situations should be considered carefully, which this paper has tried to discuss. Considering all these 24 types of cases together, the linear form of the sign realized jump intensity, the negative realized jump arrival rate and the realized jump standard deviation are able to explain some part of the observable cross-sectional returns.

## Conclusion

For risk-averse investors, jump risk, which leads to return distribution with a fat tail, can greatly affect investors’ perception about how risky a company is or how it captures the risk information. This paper applies the realized jumps to be a measure of tail risk, where daily realized jump volatility is estimated from 5-minute high-frequency trading data of Chinese A-share stocks and the stocks are divided into 25 portfolios according to their size and book-to-market ratio. Then, a non-parametric method is applied to simply and accurately calculate the positive and negative jumps and their distribution variables based on the theory of the asymmetric characteristics, including jump intensity, jump mean, jump standard deviation and jump arrival rate.

Then, we argue that the financial constraints make investors fail to effectively diversify nonsystematic risk away; hence, the tail risk should be priced, and jumps should be a good supplemental factor to explain the excess portfolio returns.

The Fama-MacBeth two-step regression test shows that both the continuous realized volatility component and the jump component have risk premia, and the jump tail risk, including the sign, negative and positive realized jump components can explain the portfolios’ excess returns as well; this sheds new light on stock return dynamics and helps us better understand and grasp the financial market rules, and such research is rare. Combining the concept of jumps with investor’s economic intuitions, such as fear of loss, hedging of the expected losses or higher risk compensation for expected losses, and the frightening expectation of catastrophic risks in the uncertain macroeconomic environment, it is beneficial for investors’ hedging risk control strategy to consider jumps. The results of this paper also tell us that we need to pay more attention to the role of jumps, particularly large negative jumps.

## Supporting information

S1 Text Descriptive statistics of the jump components for the other 20 portfolios. ***, **, * represent the 1%, 5%, and 10% significance levels of the Jarque-Bera Test, respectively.
(DOCX)

S1 Table Descriptive statistics of the sign jump intensity (sign_size) for Fama-French portfolios. ***, **, * represent the 1%, 5%, and 10% significance levels of the Jarque-Bera Test, respectively.
(DOCX)
