# Supplementary material for: Sign realized jump risk and the cross-section of stock returns: Evidence from China's stock market
Source: PLoS One. 2017 Aug 3;12(8):e0181990. doi: 10.1371/journal.pone.0181990 (PMC5542663; doi:10.1371/journal.pone.0181990)
Supplement: S1 Text — ***, **, * represent the 1%, 5%, and 10% significance levels of the Jarque-Bera Test, respectively. (DOCX) [file pone.0181990.s001.docx]

**Table A. Descriptive Statistics of the Jump Components for s2b1-s2b5**

| **Panel A the Negative Jump Components** | | | | | | | |
| --- | --- | --- | --- | --- | --- | --- | --- |
| **Portfolios** | **NN** | **NP** | **N_Size** | **N_Mean** | **N_Arr** | **N_Std** | **BV** |
| s2b1 | 1.0452^***^ | 2.9821^***^ | 0.0219^***^ | 0.0132^***^ | 0.0528^***^ | 0.0111^***^ | 0.0217^***^ |
| s2b2 | 1.0613^***^ | 2.7899^***^ | 0.0245^***^ | 0.0146^***^ | 0.0537^***^ | 0.0123^***^ | 0.0242^***^ |
| s2b3 | 1.1311^***^ | 2.7724^***^ | 0.0262^***^ | 0.0153^***^ | 0.0576^***^ | 0.0127^***^ | 0.0260^***^ |
| s2b4 | 1.1133^***^ | 2.7580^***^ | 0.0268^***^ | 0.0159^***^ | 0.0567^***^ | 0.0133^***^ | 0.0280^***^ |
| s2b5 | 1.1682^***^ | 3.0034^***^ | 0.0294^***^ | 0.0167^***^ | 0.0614^***^ | 0.0138^***^ | 0.0290^***^ |
| **Panel B the Positive Jump Components** | | | | | | | |
| **Portfolios** | **NN** | **NP** | **P_Size** | **P_Mean** | **P_Arr** | **P_Std** | **BV** |
| s2b1 | 1.0452^***^ | 2.9821^***^ | 0.0590^***^ | 0.0188^***^ | 0.1502^***^ | 0.0031^***^ | 0.0217^***^ |
| s2b2 | 1.0613^***^ | 2.7899^***^ | 0.0618^***^ | 0.0210^***^ | 0.1408^***^ | 0.0034^***^ | 0.0242^***^ |
| s2b3 | 1.1311^***^ | 2.7724^***^ | 0.0654^***^ | 0.0220^***^ | 0.1407^***^ | 0.0037^***^ | 0.0260^***^ |
| s2b4 | 1.1133^***^ | 2.7580^***^ | 0.0700^***^ | 0.0234^***^ | 0.1424^***^ | 0.0040^***^ | 0.0280^***^ |
| s2b5 | 1.1682^***^ | 3.0034^***^ | 0.0782^***^ | 0.0233^***^ | 0.1572^***^ | 0.0038^***^ | 0.0290^***^ |

| **Table B. Descriptive Statistics of the Jump Components for s3b1-s3b5** | | | | | | | |
| --- | --- | --- | --- | --- | --- | --- | --- |
| **Panel A the Negative Jump Components** | | | | | | | |
| **Portfolios** | **NN** | **NP** | **N_Size** | **N_Mean** | **N_Arr** | **N_Std** | **BV** |
| s3b1 | 1.1150^***^ | 3.2837^***^ | 0.0223^***^ | 0.0130^***^ | 0.0564^***^ | 0.0107^***^ | 0.0213^***^ |
| s3b2 | 1.0705^***^ | 2.7619^***^ | 0.0250^***^ | 0.0149^***^ | 0.0543^***^ | 0.0125^***^ | 0.0244^***^ |
| s3b3 | 1.1371^***^ | 2.6836^***^ | 0.0266^***^ | 0.0157^***^ | 0.0575^***^ | 0.0131^***^ | 0.0252^***^ |
| s3b4 | 1.1197^***^ | 2.6054^***^ | 0.0285^***^ | 0.0163^***^ | 0.0569^***^ | 0.0135^***^ | 0.0277^***^ |
| s3b5 | 1.1637^***^ | 2.8208^***^ | 0.0298^***^ | 0.0174^***^ | 0.0590^***^ | 0.0144^***^ | 0.0295^***^ |
| **Panel B the Positive Jump Components** | | | | | | | |
| **Portfolios** | **NN** | **NP** | **P_Size** | **P_Mean** | **P_Arr** | **P_Std** | **BV** |
| s3b1 | 1.1150^***^ | 3.2837^***^ | 0.0603^***^ | 0.0183^***^ | 0.1655^***^ | 0.0034^***^ | 0.0213^***^ |
| s3b2 | 1.0705^***^ | 2.7619^***^ | 0.0616^***^ | 0.0213^***^ | 0.1398^***^ | 0.0036^***^ | 0.0244^***^ |
| s3b3 | 1.1371^***^ | 2.6836^***^ | 0.0620^***^ | 0.0217^***^ | 0.1354^***^ | 0.0038^***^ | 0.0252^***^ |
| s3b4 | 1.1197^***^ | 2.6054^***^ | 0.0649^***^ | 0.0225^***^ | 0.1321^***^ | 0.0037^***^ | 0.0277^***^ |
| s3b5 | 1.1637^***^ | 2.8208^***^ | 0.0768^***^ | 0.0250^***^ | 0.1447^***^ | 0.0047^***^ | 0.0295^***^ |

| **Table C. Descriptive Statistics of the Jump Components for s4b1-s4b5** | | | | | | | |
| --- | --- | --- | --- | --- | --- | --- | --- |
| **Panel A the Negative Jump Components** | | | | | | | |
| **Portfolios** | NN | NP | N_Size | N_Mean | N_Arr | N_Std | BV |
| s4b1 | 1.0669^***^ | 3.0858^***^ | 0.0218^***^ | 0.0133^***^ | 0.0538^***^ | 0.0112^***^ | 0.0215^***^ |
| s4b2 | 1.0657^***^ | 2.6891^***^ | 0.0244^***^ | 0.0146^***^ | 0.0537^***^ | 0.0122^***^ | 0.0245^***^ |
| s4b3 | 1.0588^***^ | 2.6722^***^ | 0.0257^***^ | 0.0152^***^ | 0.0537^***^ | 0.0128^***^ | 0.0252^***^ |
| s4b4 | 1.1713^***^ | 2.6953^***^ | 0.0282^***^ | 0.0160^***^ | 0.0595^***^ | 0.0132^***^ | 0.0271^***^ |
| s4b5 | 1.2094^***^ | 2.6908^***^ | 0.0321^***^ | 0.0178^***^ | 0.0616^***^ | 0.0146^***^ | 0.0286^***^ |
| **Panel B the Positive Jump Components** | | | | | | | |
| **Portfolios** | NN | NP | P_Size | P_Mean | P_Arr | P_Std | BV |
| s4b1 | 1.0669^***^ | 3.0858^***^ | 0.0604^***^ | 0.0193^***^ | 0.1552^***^ | 0.0035^***^ | 0.0215^***^ |
| s4b2 | 1.0657^***^ | 2.6891^***^ | 0.0607^***^ | 0.0211^***^ | 0.1356^***^ | 0.0037^***^ | 0.0245^***^ |
| s4b3 | 1.0588^***^ | 2.6722^***^ | 0.0614^***^ | 0.0218^***^ | 0.1353^***^ | 0.0036^***^ | 0.0252^***^ |
| s4b4 | 1.1713^***^ | 2.6953^***^ | 0.0649^***^ | 0.0229^***^ | 0.1371^***^ | 0.0043^***^ | 0.0271^***^ |
| s4b5 | 1.2094^***^ | 2.6908^***^ | 0.0705^***^ | 0.0244^***^ | 0.1379^***^ | 0.0044^***^ | 0.0286^***^ |

**Table D. Descriptive Statistics of the Jump Components for s5b1-s5b5**

| **Panel A the Negative Jump Components** | | | | | | | |
| --- | --- | --- | --- | --- | --- | --- | --- |
| **Portfolios** | **NN** | **NP** | **N_Size** | **N_Mean** | **N_Arr** | **N_Std** | **BV** |
| s5b1 | 0.9931^***^ | 3.0611^***^ | 0.0200^***^ | 0.0120^***^ | 0.0499^***^ | 0.0101^***^ | 0.0188^***^ |
| s5b2 | 1.0381^***^ | 2.5756^***^ | 0.0239^***^ | 0.0142^***^ | 0.0526^***^ | 0.0119^***^ | 0.0217^***^ |
| s5b3 | 1.0924^***^ | 2.5840^***^ | 0.0263^***^ | 0.0153^***^ | 0.0553^***^ | 0.0127^***^ | 0.0229^***^ |
| s5b4 | 1.1779^***^ | 2.3515^***^ | 0.0552^***^ | 0.0208^***^ | 0.1195^***^ | 0.0036^***^ | 0.0246^***^ |
| s5b5 | 1.2556^***^ | 2.4039^***^ | 0.0331^***^ | 0.0181^***^ | 0.0637^***^ | 0.0148^***^ | 0.0256^***^ |
| **Panel B the Positive Jump Components** | | | | | | | |
| **Portfolios** | **NN** | **NP** | **P_Size** | **P_Mean** | **P_Arr** | **P_Std** | **BV** |
| s5b1 | 0.9931^***^ | 3.0611^***^ | 0.0575^***^ | 0.0186^***^ | 0.1536^***^ | 0.0034^***^ | 0.0188^***^ |
| s5b2 | 1.0381^***^ | 2.5756^***^ | 0.0555^***^ | 0.0197^***^ | 0.1306^***^ | 0.0031^***^ | 0.0217^***^ |
| s5b3 | 1.0924^***^ | 2.5840^***^ | 0.0569^***^ | 0.0201^***^ | 0.1310^***^ | 0.0039^***^ | 0.0229^***^ |
| s5b4 | 1.1779^***^ | 2.3515^***^ | 0.0552^***^ | 0.0208^***^ | 0.1195^***^ | 0.0036^***^ | 0.0246^***^ |
| s5b5 | 1.2556^***^ | 2.4039^***^ | 0.0638^***^ | 0.0234^***^ | 0.1225^***^ | 0.0045^***^ | 0.0256^***^ |

^***^, ^**^, ^*^ represent the 1%, 5%, and 10% significance levels of the Jarque-Bera Test, respectively.
