# Supplementary material for: Sign realized jump risk and the cross-section of stock returns: Evidence from China's stock market
Source: PLoS One. 2017 Aug 3;12(8):e0181990. doi: 10.1371/journal.pone.0181990 (PMC5542663; doi:10.1371/journal.pone.0181990)
Supplement: S1 Table — ***, **, * represent the 1%, 5%, and 10% significance levels of the Jarque-Bera Test, respectively. (DOCX) [file pone.0181990.s002.docx]

**Table S1. Descriptive Statistics of the Sign Jump Intensity (Sign_Size) for Fama-French Portfolios**

| **Sign_Size** | **b1** | **b2** | **b3** | **b4** | **b5** |
| --- | --- | --- | --- | --- | --- |
| **s1** | 0.0362^***^ | 0.0369^***^ | 0.0397^***^ | 0.0422^***^ | 0.0537^***^ |
| **s2** | 0.0371^***^ | 0.0373^***^ | 0.0009^***^ | 0.0432^***^ | 0.0486^***^ |
| **s3** | 0.0380^***^ | 0.0366^***^ | 0.0355^***^ | 0.0365^***^ | 0.0470^***^ |
| **s4** | 0.0386^***^ | 0.0363^***^ | 0.0357^***^ | 0.0366^***^ | 0.0384^***^ |
| **s5** | 0.0376^***^ | 0.0316^***^ | 0.0306^***^ | 0.0262^***^ | 0.0307^***^ |

^***^, ^**^, ^*^ represent the 1%, 5%, and 10% significance levels of the Jarque-Bera Test, respectively.
